# Phenolic Constituents, Antioxidant and Preliminary Antimycoplasmic Activities of Leaf Skin and Flowers of *Aloe vera* (L.) Burm. f. (syn. *A. barbadensis* Mill.) from the Canary Islands (Spain)

**DOI:** 10.3390/molecules18054942

**Published:** 2013-04-26

**Authors:** Aroa López, Miguel Suárez de Tangil, Orestes Vega-Orellana, Ana S. Ramírez, Milagros Rico

**Affiliations:** 1 Departamento de Química, Universidad de Las Palmas de Gran Canaria, Unidad Asociada al CSIC, Campus de Tafira, 35017 Las Palmas de Gran Canaria, Canary Islands, Spain; 2 Unidad de Epidemiología y Medicina Preventiva, Facultad de Veterinaria, Universidad de Las Palmas de Gran Canaria, C/Trasmontaña s/n, Arucas, 35413, Islas Canarias, Spain

**Keywords:** *Aloe vera*, leaf skin, phenolic constituents, antioxidant activity, antimycoplasmic activity

## Abstract

The methanol extracts of leaf skins and flowers of *Aloe vera* from the Canary Islands were analyzed for their phenolic profiles and screened for their antioxidant and antimycoplasmic activities. The use of reversed phase high performance liquid chromatography (RP-HPLC) allowed the identification of 18 phenolic constituents. Leaf skin extracts were characterized by the abundance of catechin, sinapic acid and quercitrin. Gentisic acid, epicatechin and quercitrin were the most prominent phenolic compounds of the flowers. The *in vitro* antioxidant activities determined by using the 1,1-diphenyl-2-picrylhydrazyl (DPPH) and ferric antioxidant reducing power (FRAP) assays revealed that both extracts exhibited antioxidant activity, being the leaf skin extract the most active fraction. The leaf skin extract was also found to be active against the microbial strains tested. Therefore, *A. vera* extracts from leaf skin and flowers can be considered as good natural antioxidant sources.

## 1. Introduction

The botanical name of *Aloe vera* is *Aloe barbadensis* Miller. It belongs to the Asphodelaceae (Liliaceae) family, and is a shrubby or arborescent, perennial, xerophytic, succulent pea-green colored plant [1]. The origins of these plants are the dry regions of Africa, Asia, and Southern Europe, especially in the Mediterranean regions. In the Canary Islands, *Aloe vera* plants naturally grow anywhere and everywhere and there is a considerable generally shared belief in the beneficial action of the gel among the population, estimated to be one of the few botanical medications in widespread domestic use. In folk medicine, the brown juice has been used traditionally for its purgative effects, and the fresh leaf gel in cosmetics and nutraceutical formulations [2]. Among the purported benefits of *Aloe vera* not supported by experimental or clinical data are the following: treatment of acne, haemorrhoids, psoriasis, anemia, glaucoma, petit ulcer, tuberculosis, blindness, seborrhoeic dermatitis, and fungal infections [2]. Several reports have demonstrated the antioxidant, antinociceptive and anti-inflammatory activities of the aloe species [3,4]. In addition, recent studies have shown the anti-cancer effect of aloe-emodin, an anthraquinone compound present in the leaves of *Aloe vera* [5]. Studies of the *in vitro* antimicrobial properties of the ethanolic extract of *Aloe vera* leaf gel revealed that it was active against most of the studied pathogenic bacteria and fungi, even at very low doses [6]. The list of different illnesses and conditions aided by the use of *Aloe vera* is indeed impressive, covering everything from burns and slight infections to extremely serious medical conditions. Several reviews have focused on the main scientific discoveries on *Aloe vera* reported over the last three decades [2,7,8,9,10,11,12]. These reviews deal with the botany, the chemical properties, the gel stabilization technique, the biological functions, and the current uses and applications of *Aloe vera* (mainly focusing on the exudate and gel of the *Aloe vera* leaves).

Plant polyphenols have been implicated in diverse functional roles, including plant resistance against microbial pathogens and animal herbivores such as insects (antibiotic and antifeeding actions), protection against solar radiation, besides reproduction, nutrition, and growth [13]. Phenolic compounds have also been reported to prevent diseases resulting from oxidative stress [14,15,16].

Screening of the phytochemical (qualitative and quantitative) analysis of the *Aloe vera* leaf (leaf skin and gel) showed that almost all of the chemical constituents are present: tannin, phlobatannins, saponin, flavonoids, steroids, terpenoids, and cardiac glycosides anthroquinones, which are used for medicinal purposes [17]. Phenolic compounds are the second major substances found in *Aloe vera*. The main active constituent of the *Aloe vera* plant extract is aloine, an anthraquinone heteroside [18]. Several papers have also been published that focus on the identification of the main phenolic compounds from the gel and leaf exudate of *Aloe*. Okamura *et al.* developed a procedure for determination of aloesin, 2'-*O*-feruloylaloesin, aloeresin A, barbaloin, isobarbaloin, aloenin, aloe-emodin, 8-C-glucosyl-7-*O*-methyl-(*S*)-aloesol, isoaloeresin D and aloeresin E, which are phenolic constituents of aloe [19]. Thirteen phenolic compounds from *Aloe barbadensis (syn. A. vera)* and *A. arborescens* were identified and quantified: aloesin, 8-*C*-glucosyl-7-*O*-methyl-(*S*)-aloesol, neoaloesin A, 8-*O*-methyl-7-hydroxyaloin A and B, 10-hydroxyaloin A, isoaloeresin D, aloin A and B, aloeresin E and aloe-emodin from *A. barbadensis;* and aloenin, aloenin B, 10-hydroxyaloin A, aloin A and B, and aloe-emodin from *A. arborescens* [20]. A mixture of phenolic compounds, mainly anthrones (aloenin, aloenin B, isobarbaloin, barbaloin and other aloin derivatives from *Aloe secundiflora* (Aloeaceae) has been determined from the leaf exudate [21]. So far, little attention has been paid to the flowers or the leaf skin of the *Aloe*. Previous studies have suggested using *Aloe vera* flowers for phytotherapeutical purposes due to the presence of some polyphenols [22].

The aim of this study was to determine the differences in the phenolic profile of the methanol extracts derived from the leaf skin and flowers of *Aloe vera* (L.) Burm. f. (syn. *A. barbadensis* Mill.) from the Canary Islands to investigate the potential of the flowers and the leaf skin for uses in the health food industry, as well as in pharmaceuticals. As a result eighteen phenolic components were identified and quantified by reverse phase-high performance liquid chromatography (RP-HPLC). The antioxidant activities of the extracts were studied, as well as the preliminary *in vitro* susceptibility of some mycoplasma strains. Mollicutes, trivially known as mycoplasmas, are phylogenetically related to the Gram-positive branch of the eubacteria and can be divided into five phylogenetic groupings, including the *anaeroplasma, asteroleplasma, spiroplasma, pneumoniae*, and *hominis* groups [23]. Mycoplasmas are commensals or parasites on vertebrate, insect, and plant hosts, representing many significant pathogens in human and veterinary medicine. They are bacteria characterized by their lack of a cell wall and for their small genomes and highly structural and functional simplicity. Besides, they do not synthesize nucleotides or amino acids, express an unusual form of RNA polymerase, and certain species produce atypical ribosomes. All these characteristics make them naturally resistant to many antibiotics, reducing treatment options to tetracyclines, macrolides, and fluoroquinolones. Therefore, they represent magnificent targets for anti-microbe testing [24].

## 2. Results and Discussion

### 2.1. Determination of the Phenolic Profile by HPLC

The presence of polyphenols in the extracts was confirmed by comparing retention times (RT) and overlapping UV spectra with those of standard compounds. The phenolic compounds sinapic acid (RT: 6.9 min), quercitrin (RT: 7.6 min), kaempferol (RT: 10.8 min) and apigenin (RT: 11.3 min) were well resolved. Limits of detection (LOD) and limits of quantification (LOQ) were estimated from signal-to noise-ratio of the individual peaks, assuming a minimum detectable signal-to-noise level of 3 and 10 respectively [25]. The LODs were found to be in the range of 0.032–0.127 μg·mL^−1^ and the LOQs were observed in the range of 0.106–0.355 μg·mL^−1^ (Table 1). This indicated that the proposed method offers adequate sensitivity for the quantification of polyphenols. Reproducibility, expressed as the relative standard deviation (RSD), was obtained by analysing six replicate samples containing 20 μg·mL^−1^ of each of the four compounds. The accuracy was expressed as the recovery of standard compounds added to the pre-analysed sample [26]. The results are summarized in Table 1. The phenolic compounds gallic acid, protocatechuic acid, catechin, vanillic acid, epicatechin and syringic acid, chlorogenic acid, gentisic acid, caffeic acid, *p-*coumaric acid and ferulic acid, rutin, myricetin, and quercetin were quantified according to a previously reported method [27].

**Table 1:** Method validation data for the quantitative determination of four phenolic compounds using RP-HPLC.

Compounds	Regression equation (r)	LOD ^a^ μg·mL^−1^	LOQ ^a^ μg·mL^−1^	Recovery ^b^ (%)	RSD ^c^ (%)
Sinapic acid	y = 34493x − 25074 (0.9988)	0.10661	0.3554	118 ± 3	2.57
Quercitrin	y = 88302x − 20416 (0.9976)	0.03640	0.1235	116 ± 4	3.69
Kaempferol	y = 15436x − 28177 (0.9998)	0.09775	0.3258	105 ± 8	7.61
Apigenin	y = 20306x − 67494 (0.9993)	0.03196	0.1065	99 ± 3	2.66

^a^ Detection limits are calculated as signal to noise ratio of ten times; ^b^ Means ± standard deviation of three measurements; ^c^ Reproducibility was obtained by analyzing six replicate samples containing 20 μg·mL^−1^ for every standard.

The proposed polyphenols were identified in the extracts, except for gallic acid, which was only detected in the flower extract. In addition, quercetin was only detected in the leaf skin extract. The results here evidenced that catechin, sinapic acid, gentisic acid and epicatechin were the most abundant compounds of those under study and their mix ratio changed depending on the source of the extract (aloe leaf skin or flowers). Catechin and sinapic acid were most abundant in the leaf skin extract and gentisic acid and epicatechin were predominant in the flower extract (Table 2).

**Table 2:** The polyphenol contents in aloe extracts presented as average values ± standard deviation of two measurements.

Phenolic compound	Leaf skin ^a^	Flowers ^a^
Sinapic acid	54 ± 3	15.0 ± 0.6
Quercitrin	23 ± 1	31.9 ± 0.5
Kaempferol	4.03 ± 0.03	2.86 ± 0.01
Apigenin	3.3 ± 0.4	3.03 ± 0.00
Gallic acid	nd ^b^	12.6 ± 0.2
Protocatechuic	1.1 ± 0.0	0.57 ± 0.02
Catechin	95 ± 3	7.6 ± 0.2
Vanillic acid	2.30 ± 0.04	0.8 ± 0.1
Epicatechin	16.2 ± 0.7	58.0 ± 0.1
Syringic acid	4.9 ± 0.5	5.0 ± 0.3
Chlorogenic acid	7.8 ± 0.2	2.8 ± 0.2
Gentisic acid	6.0 ± 0.3	101 ± 2
Caffeic acid	4.9 ± 0.1	9.3 ± 0.1
Coumaric acid	0.8 ± 0.0	7.6 ± 0.4
Ferulic acid	7.9 ± 0.4	3.1 ± 0.1
Rutin	22.3 ± 2	11.6 ± 0.2
Miricetin	19.6 ± 0.7	1.76 ± 0.02
Quercetin	34.4 ± 2	nd ^b^
Sum	307.5	274.5

^a^ mg per 100 gram of freeze-dried aloe material; ^b^ nd indicates not detected.

The chemistry of the aloe plant has been studied for many years from a number of viewpoints [3,4,5,6,7,8,9,10,11,12]. Interest has centered on the parenchyma gel and its well-known therapeutic properties. Previous studies regarding the content of polyphenols in the leaf skin were not found. However, various phenolic compounds such as chlorogenic, caffeic, *p*-coumaric and ferulic acids were detected in the *Aloe* flowers [22]. In the present study, we detected and quantified eighteen phenolic compounds (Table 2), confirming that the *Aloe* leaf skin and flowers are natural sources of well-known antioxidant compounds [28].

### 2.2. Radical Scavenging Activity (RSA) on DPPH and Ferric Reducing Antioxidant Power (FRAP)

The reducing ability of antioxidants on the DPPH radical was evaluated by measuring the loss of DPPH color at 515 nm after reaction with the test samples. The leaf skin extract was more active to DPPH than the flower extract (Table 3). The FRAP assay was used to study the ability of the antioxidants in these extracts to reduce the ferric iron to a ferrous form. The redox reaction is carried out at pH 3.6. At more acidic conditions than the physiological pH, the reducing capacity may be suppressed due to protonation on the phenolics. However, the same behaviour as was observed in the DPPH assay was observed instead (the extract of the leaf skin was more active than the flower extract) (Table 3).

**Table 3:** Antioxidant and antimycoplasmic activities of the aloe extracts.

Extract	RSA ^a,b^	FRAP ^a,c^	Antimycoplasmal activity ^a,d^			
			*M. mycoides capri*	*M. agalactiae*	*Acholeplasma laidlawii*	*M. gallisepticum*
Leaf skin	58.8 ± 0.4	2.4 ± 0.1	239 ± 51 ^b^	CCM	2253 ± 123	1466 ± 213
Flowers	53 ± 2	1.7 ± 0.0	-	-	-	-

^a^ Values represented mean ± standard deviation of three measurements; ^b^ % inhibition; ^c^ mmol of Fe (III) reduced to Fe(II); CCM: changes in the colony morphology around the disc - indicates no growth inhibition; ^d^ in micrometers.

In general, the literature reports that there is a relation between the content of phenolic compounds and the antioxidant properties [29]. Our results confirm the correlation between the phenolic content (calculated as the sum of the identified polyphenols) and the antioxidant activity (the extract with higher phenolic content gave the highest activities), allowing us to conclude that phenolic constituents are mainly responsible for the observed antioxidant activity in the extracts. 

### 2.3. Antimycoplasmic Activity

The four mycoplasmas were selected as representatives of four of the five mycoplasma phylogenetic groups [23]. The group without representation is the *anaeroplasma* group, composed of anaerobic commensals found in ovine and bovine rumen. Also the three mycoplasmas included in the genus *Mycoplasma* are ruminant and poultry pathogens, while the mycoplasma from the genus *Acholeplasma* is a frequent contaminant of eukaryotic cell cultures. The results of the *in vitro* susceptibility test are given in Table 3. The concentration of the extracts is slightly higher than the ones used by Al-Momani *et al.* [30]. Antimycoplasmic activity was only found in the leaf skin extract (from simple changes in the colony morphology (CCM) as occurred in the case of *Mycoplasma agalactiae (M. agalactiae)* or in greater inhibition zones (*Acholeplasma laidlawii* and *Mycoplasma gallisepticum*))*.* Further studies have to be undertaken to validate and extend these observations over a wider selection of bacteria and fungi in order to determine minimum inhibitory concentrations (MIC) of these extracts. 

The extract from the flowers did not show antimycoplasmic activity against the bacterial strains tested in the present study, but might express strong properties against other test organism or other kind of properties depending on its composition in phenolic compounds. Rodríguez Vaquero *et al.* reported that bacterial species exhibited different sensitivities towards the different concentrations of pure phenolic compounds [31]. *Escherichia coli* was the most sensitive bacterium and *Flavobacterium sp*. was resistant against all phenolic compounds tested. The activity of the extracts is not only dependent on the concentration of the phenolic compounds but also on the structure and nature of the compounds [32]. Several reports have suggested that the biological activity of the extracts is also dependent on the interaction among the phenolic compounds [33,34]. Tafesh *et al.* [35] reported that hydroxytyrosol at 400 µg·mL^−1^ caused growth inhibition of the four bacterial isolate gram-positive (*Streptococcus pyogenes and Staphylococcus aureus*) together with the gram-negative (*Escherichia coli* and *Klebsiella pneumoniae*), while gallic acid at 200 and 400 µg mL^−1^ inhibited the growth of the *S. aureus* and *S. pyogenes* strains, respectively (no growth inhibition was observed for the gram-negative bacteria). However, the combination of both compound gallic acid and hydroxytyrosol at lower concentrations (100 and 200 μg·mL*^−^*^1^, respectively) caused a complete inhibition of the four bacterial strains. Gao *et al.* [36] reported that four phenolic compounds of those under study (vanillic acid, protocatechuic acid, ferulic acid and caffeic acid) exerted additive and synergistic inhibition effects on the growth of *Microcystis aeruginosa* depending on the mix ratios. In said study, the authors concluded that the profile of the phenolic compounds in a mixture and their mix ratios determine the joint action of the compounds, with either synergistic, antagonistic and/or additive effects. The efficacy of a combination of different phenolic compounds structures might be greater than that of other combinations on a kind of activity. Therefore, various different kinds of activities of a phenolic mixture may also depend on the mix ratios, where the mixture may be more active in contributing to UV-B protection or warding off microbial infection or protecting the plants from herbivores. The differences in the phenolic profile in the present work may be the result of the involvement of these compounds in different functional roles in the flowers from in the leaf skin. Further research is required to study the joint action of the phenolic compounds identified in the extracts and the influence of the mix ratio of said phenolic compounds on the kind and intensity of the mix activity.

## 3. Experimental

### 3.1. Chemicals

Methanol (of HPLC grade), ferric chloride (FeCl_3_·6H_2_O), ferrous sulphate (FeSO_4_·7H_2_O) and glacial acetic acid were obtained from Panreac (Barcelona, Spain) with formic acid and sodium acetate provided by Merck (Darmstadt, Germany) of analytical quality. The 1,1-Diphenyl-2-picrylhydrazyl (DPPH) and 2,4,6-tri(2-pyridyl)-1,3,5-triazine (TPTZ) were from Sigma-Aldrich Chemie (Steinheim, Germany).

The antimicrobial activity of the plant extracts was tested using susceptibility test disks (Oxoid, CT0998B, Ø = 5 mm). Polyphenol standards of gallic acid, protocatechuic acid, chlorogenic acid, (−) epicatechin, quercetin, myricetin, ferulic acid, *p-*coumaric acid, vanillic acid, syringic acid, (+) catechin, sinapic acid, quercitrin, kaempferol and apigenin were purchased from Sigma-Aldrich Chemie; rutin and gentisic and caffeic acids were supplied by Merck (Hohenbrunn, Germany).

### 3.2. Mycoplasmas

Four types of strains of mollicutes: *Mycoplasma mycoides* subsp. Capri (Y-goat) (*M. mycoides* Capri), *Mycoplasma* *agalactiae* (PG2) (*M. agalactiae*), *Acholeplasma* (A.) *laidlawii* (PG8) (*A. laidlawii*) and *Mycoplasma gallisepticum* (PG31) (*M. gallisepticum*) were used in this study with the former two cultivated in PH medium [37] and the latter two in SP4-II medium [38]. The mycoplasmas were cultured under aerobic conditions at 37 °C.

### 3.3. Plant Material

The *Aloe vera* leaves and flowers were collected fresh in February 2010. The plant was identified in the Herbarium of the Viera y Clavijo Botanical Gardens in Gran Canaria where a voucher specimen was deposited (LPA: 27058-27060). A voucher specimen was deposited in the Herbarium of the Viera y Clavijo Botanical Gardens in Gran Canaria (LPA: 27058-27060). Soon after collection, the skin of the leaves and the flowers were separated, shaken and frozen. The leaf skin was separated and cleaned with a knife. The frozen samples were then freeze-dried and pulverized into powder using a blender (Moulinex, 600 W, Ecully Cedex, France) and were subsequently kept in the dark at −20 °C under nitrogen.

### 3.4. Preparation of Aloe Extracts

Freeze-dried plant material (10.0 g of leaf skin and 10.0 g of flowers separately) was extracted with solvent (175 mL) in a Soxhlet extractor for 3 h. The extraction was repeated four times, using the same plant material, but different solvents: hexane, acetone, ethanol and methanol were used consecutively. Several studies have reported that high levels of polyphenols may be associated with the use of polar solvents in the extraction [27,39]. Therefore, hexane, acetone and ethanol extracts were discarded. After extraction, 10 mL of methanol extract was reserved for the antioxidant activity assays. 

To prepare the samples for the antimycoplasmic activity determination, the methanol extract was evaporated under reduced pressure to give semi-solid residues. In order to avoid problems related to the use of plant extracts, their solubility and the use of solubilising agents [40], the only product used for solubilising the residues from methanol extracts was ultra-pure sterile water. Working concentrations were 111 mg·mL^−1^ for flower residues and 112 mg·mL^−1^ for the leaf skin residues.

### 3.5. Free Radical Scavenging Activity on DPPH

The reducing ability of the antioxidants on the DPPH radical was evaluated by measuring the loss of 1,1-diphenyl-2-picrylhydrazyl (DPPH) colour at 515 nm after reaction with test extracts [41]. The sample solution (100 μL of extracts) was rapidly mixed with 1 mL of a solution of 0.2 mM DPPH. After 30 min incubation in darkness at ambient temperature (23 °C), the reduction of the DPPH radical was measured by monitoring the decline in absorbance (Abs) against a methanol blank at 515 nm using a Shimadzu 1700 UV-Vis spectrophotometer. The percentage inhibition was calculated by application of the equation: RSA = 100 (1 − Abs in the presence of sample/Abs in the absence of sample).

### 3.6. Ferric Reducing Antioxidant Power Assay (FRAP Assay)

Reducing power was determined according to [42]. This method is based on the reduction of Fe^3+^ to Fe^2+^, which is recorded by measuring the formation of a blue coloured Fe^2+^-tripyridyltriazine compound from the colourless oxidized Fe^3+^ form via the action of electron-donating antioxidants. The FRAP reagent consists of 300 mM acetate buffer (3.1 g sodium acetate + 16 mL glacial acetic acid, made up to 1 litre with distilled water; pH = 3.6), 10 mM TPTZ in 40 mM HCl and 20 mM FeCl_3_·6H_2_O in a ratio of 10:1:1.

The extract (50 μL) was added to 1.5 mL freshly prepared and pre-warmed (37 °C) FRAP reagent. The mixture was incubated at 37 °C for 10 min and the absorbance was measured against a reagent blank (1.5 mL FRAP reagent + 50 μL distilled water) at 593 nm. A standard curve of Fe(II) was constructed over the concentration range of 0.1 mM to 2.0 mM. The results were determined by the regression equation of the curve (Abs = 0.00221[Fe(II)] + 0.02464, r = 0.9998) and expressed as µmol ferric ions reduced to ferrous form.

### 3.7. Determination of the Phenolic Profile by RP-HPLC

To prepare the samples for the HPLC quantification, the freeze-dried plant material (50 mg) and 2 mL of methanol were mixed and homogenized using a vortex for 30 s. The mixture was stirred in a rotator (SB 3, Stuart, Staffordshire, UK) for 60 min at room temperature in darkness. After centrifugation at 7,000 × *g* for 20 min at 4 °C, the supernatant was collected and evaporated. The dry residue and 0.5 mL of water were mixed and filtered through a 45 μm nylon syringe filter prior to injection.

Chromatographic analysis was performed on a Varian ProStar 210 system, equipped with a vacuum degasser, a binary pump, a thermostat column compartment and a diode array detector (DAD), connected to ChemStation software. The separation was performed with a reverse-phase Pursuit XRs C18 (250 mm × 4.6 mm, 5 micrometers (μm)) column and a Pursuit XRs C18 (10 mm × 4.6 mm, 5 μm) guard column (Varian, Barcelona, Spain). A gradient system, involving two mobile phases, was used. Eluent A was water with 0.1% formic acid and eluent B, methanol. The flow rate was 1.0 mL/min, and the injection volume was 60 μL of crude extracts (rheodyne injector). The system operated at 27 °C. The elution conditions applied were: 0–4 min, linear gradient from 20% to 30% B; 4–10 min, 30% B isocratic; 10–13 min, linear gradient from 30% to 50% B; 13–15 min, linear gradient from 50% to 80% B and finally, washing and re-conditioning of the column. Monitoring was set at 254 nm for quantification. 

*Method 1*: to quantify the compounds sinapic acid, quercitrin, kaempferol and apigenin in the extracts, five different concentrations of the analytes were injected in triplicate. The calibration curves were constructed by plotting the peak areas *versus* the concentration of each analyte. The linearity was assessed by linear regression analysis, which was calculated by the least squares method. Each point on the calibration plot was the mean of two area measurements. All correlation coefficients were over 0.9976 (Table 2). The wavelength was fixed at 254 nm for quantification. The selectivity of the method was determined by analysis of standard compounds and samples. The peaks of polyphenols were identified by comparing their retention times (RT) and overlaying of UV spectra with those of standard compounds. 

*Method 2*: the phenolic compounds gallic acid, protocatechuic acid, catechin, vanillic acid, epicatechin and syringic acid, chlorogenic acid, gentisic acid, caffeic acid, *p-*coumaric acid and ferulic acid, rutin, myricetin, and quercetin were quantified in line with a previously reported method [27]. Briefly, eluent A was Milli-Q water with 0.1% formic acid and eluent B was methanol. The elution conditions applied were: 0–5 min, 20% B isocratic; 5–30 min, linear gradient from 20% to 60% B; 30–35 min, 60% B isocratic; 35–40, linear gradient from 60% to 20% B and finally, washing and reconditioning of the column. Each standard was individually tested to determine its retention times (RT) as follows: gallic acid (RT: 5.3 min), protocatechuic (RT: 10.0 min), catechin (RT: 12.7 min), chlorogenic acid (RT: 14.9 min), gentisic acid (RT: 17.1 min), vanillic acid (RT: 17.7 min), epicatechin (RT: 17.9 min), caffeic acid (17.9 min), syringic acid (RT: 18.9 min), coumaric acid (RT: 23.4 min), rutin (RT: 28.1 min), ferulic acid (RT: 24.3 min), myricetin (RT: 30.6 min), and quercetin (RT: 34.6 min) were well resolved. Simultaneous monitoring was set at 270 nm (gallic acid, protocatechuic acid, (+) catechin, vanillic acid, (−) epicatechin and syringic acid), 324 nm (chlorogenic acid, gentisic acid, caffeic acid, *p-*coumaric acid and ferulic acid), and 373 nm (rutin, myricetin, and quercetin) for quantification. The limits of detection (LOD) and limits of quantification (LOQ) were estimated from the signal-to noise-ratio of the individual peaks, assuming a minimum detectable signal-to-noise level of 3 and 10, respectively. The LODs were found to be in the range of 0.0003–0.1230 μg·mL^−1^ and the LOQs were observed in the range of 0.0008–0.4100 μg·mL^−1^. This indicated that the proposed method was suitably sensitive for the quantification of polyphenols. The linearity was assessed by linear regression analysis, which was calculated by the least square method. Each point on the calibration plot was the mean from two area measurements. All the correlation coefficients were no less than 0.9982. Reproducibility, expressed as the relative standard deviation (RSD), was obtained by analyzing six replicate sample RSDs values ranging from 1.91% to 5.81%. The accuracy was expressed as the recovery of standard compounds added to the pre-analyzed sample. The recovery was found to be in the range of 87.97%–115.79%.

### 3.8. Evaluation of the Antimycoplasmic Activity

The antimycoplasmic activity of all plant extracts was determined using a modified disc diffusion method as described for growth inhibition tests elsewhere [43]. A total of 25 μL of each extract was tested in the disc diffusion assay against four strains of mycoplasmas. The plates were subsequently incubated and examined daily for colonies (1–2 days) under the conditions described above. Organisms were considered resistant when their growth was not inhibited by the 25 µL extract-impregnated wafers (5-mm sterilized filter paper discs). The presence of a zone of inhibition, as well as any changes in the colony morphology or in the colony concentration, was considered to be indicative of antimycoplasmic activity. The inhibition zones were measured in µm using an optical microscope Olympus CKX41 (Olympus, Hamburg, Germany), with a digital camera ProgRes C12 plus (Jenoptik, Jena, Germany) inserted, and using ProgRes® Image Capture Software for the measurements. Each antimycoplasmal assay was performed at least in triplicate and inhibition zones were measured at least three times per well, at perpendicular angles. Mean values and standard deviations (SD) were registered and calculated as mean ± SD to the effects of this study. Filter discs impregnated with 25 μL of Tilmicosin (0.4 μg/mL) were used as positive control for antimicrobial activity and impregnated with 25 μL of distilled sterilised water were used as negative controls.

## 4. Conclusions

Phenolic compounds are active principles of medicinal plants and exhibit pharmacological effects that contribute towards human health. The presence of polyphenols in these extracts and their antioxidant and antimycoplasmic activities offer motivating results that suggest the potential feasibility of using *Aloe vera* leaf skin and flowers in the health food and general food industries, or as an ingredient in other products, as well as their possible applications in the pharmaceutical industry.
